# Crossed functional specialization between the basal ganglia and cerebellum during vocal emotion decoding: Insights from stroke and Parkinson’s disease

**DOI:** 10.3758/s13415-022-01000-4

**Published:** 2022-04-26

**Authors:** Marine Thomasson, Damien Benis, Philippe Voruz, Arnaud Saj, Marc Vérin, Frédéric Assal, Didier Grandjean, Julie Péron

**Affiliations:** 1 Department of Psychology, Clinical and Experimental Neuropsychology Laboratory, University of Geneva, Geneva, Switzerland; 2 Department of Psychology and Swiss Centre for Affective Sciences, Neuroscience of Emotion and Affective Dynamics Laboratory, University of Geneva, Geneva, Switzerland; 3 Department of Neurology, Cognitive Neurology Unit, University Hospitals of Geneva, Geneva, Switzerland; 4 Department of Psychology, University of Montreal, Montreal, QC Canada; 5 Behavior and Basal Ganglia Research Unit, University of Rennes 1-Rennes University Hospital, Rennes, France; 6 Neurology Department, Pontchaillou Hospital, Rennes University Hospital, Rennes, France; 7 Faculty of Medicine, University of Geneva, Geneva, Switzerland

**Keywords:** Cerebellum, Parkinson’s disease, Basal ganglia, Vocal emotion

## Abstract

There is growing evidence that both the basal ganglia and the cerebellum play functional roles in emotion processing, either directly or indirectly, through their connections with cortical and subcortical structures. However, the lateralization of this complex processing in emotion recognition remains unclear. To address this issue, we investigated emotional prosody recognition in individuals with Parkinson’s disease (model of basal ganglia dysfunction) or cerebellar stroke patients, as well as in matched healthy controls (*n* = 24 in each group). We analysed performances according to the lateralization of the predominant brain degeneration/lesion. Results showed that a right (basal ganglia and cerebellar) hemispheric dysfunction was likely to induce greater deficits than a left one. Moreover, deficits following left hemispheric dysfunction were only observed in cerebellar stroke patients, and these deficits resembled those observed after degeneration of the right basal ganglia. Additional analyses taking disease duration / time since stroke into consideration revealed a worsening of performances in patients with predominantly right-sided lesions over time. These results point to the differential, but complementary, involvement of the cerebellum and basal ganglia in emotional prosody decoding, with a probable hemispheric specialization according to the level of cognitive integration.

**Supplementary Information:**

The online version contains supplementary material available at 10.3758/s13415-022-01000-4.

## Introduction

There is growing evidence of the involvement of the basal ganglia (BG) and cerebellum in the recognition of vocal emotions, or *emotional prosody* (Alba-Ferrara et al., 2011; Bach et al., 2008; Frühholz et al., 2012; Grandjean et al., 2005; Imaizumi et al., 1997; Kotz et al., 2003; Kotz et al., 2013; Paulmann et al., 2008, 2009; Sidtis & Sidtis, 2003; Wildgruber et al., 2005). Based on this empirical evidence, and on theoretical propositions in the cognitive domain, authors have recently speculated about the functional specialization of these two structures in affective processing (for a review, see Pierce & Péron, 2020). In the cognitive domain, it has been suggested that the dento-thalamo-striatal pathway relays the predicted results of a given action, computed in the cerebellum, to the striatum, where the actual results of that action are ultimately assessed (forward model). Concerning the subthalamic-ponto-cerebellar pathway, one hypothesis is that it prevents the newly acquired forward models from being conveyed to the striatum (Caligiore et al., 2017). Based on this and on the internal models hypothesis (Koziol et al., 2014), some authors have suggested that during the process of recognizing emotional prosody, the BG “*enhance activity within the neural representation (i.e., habit-like chunk) corresponding to a previously reinforced emotional experience, leading to faster activation of regions downstream to reach a decision threshold and/or generate a motor response*” (Pierce & Péron, 2020, p. 607). The cerebellum, for its part, refines the cortical/BG response and recalibrates the internal model by checking whether the individual’s state varies from the expected state at any time during this processing. In the specific case of the vocal modality, the involvement of these two structures seems unsurprising, given the BG’s participation in the rhythmic aspects of speech decoding (Kotz & Schwartze, 2010) and the cerebellum’s contribution to timing and sensory acquisition (Ivry & Keele, 1989). Then, each structure would be involved in different sub-mechanisms in the processing of emotions. Moreover, each structure presumably has lateralized functional specializations, with differential roles according to the cerebral or cerebellar hemisphere. The latter proposition has been made on the basis of a corpus of clinical studies in Parkinson’s disease (PD) and cerebellar lesions.

In PD*,* setting aside the impact of both medication and deep brain stimulation (DBS) on emotion processing, the asymmetry of motor symptoms is viewed as an important clinical factor, in that it may influence the presence and severity of affective disorders (Benis et al., 2020; Stirnimann et al., 2018; Voruz et al., 2020). Studies suggest that patients with PD exhibiting predominantly left-sided (LPD) versus right-sided (RPD) motor symptoms have more severe affective deficits (Eitan et al., 2013; Garrido-Vásquez et al., 2013; Paulmann et al., 2011; Péron et al., 2017; Stirnimann et al., 2018; Ventura et al., 2012) (but Blonder et al., 1989; Buxton et al., 2013; Clark et al., 2008). Using local field potential recordings, Benis et al. (2020) observed a complex pattern of oscillatory activity in the human subthalamic nucleus (STN) in response to vocal emotions and revealed a crucial influence of disease laterality on this structure’s low-frequency oscillatory activity. Interestingly, they highlighted the possibility that the emotional prosody processing difficulties of LPD could be linked to the reduced or delayed STN responses to emotion observed in the high-frequency bands. This supports the notion of hemispheric specialization of the BG for emotional prosody recognition, with the right BG playing a dominant role in vocal emotion decoding. *Concerning the cerebellum*, one previous study suggested a greater involvement of the right posterior cerebellum (Thomasson et al., 2019) in the recognition of emotional prosody. In this study, cerebellar stroke patients rated erroneous higher Surprise when they listened to fear stimuli compared with healthy controls (HC). Furthermore, these emotional misattributions were correlated with lesions in the right cerebellar hemisphere (Lobules VIIb, VIII, and IX). To the best of our knowledge, only one study so far has investigated cerebellar hemispheric specialization for the recognition of emotional prosody (Thomasson et al., 2021). Results revealed impairment of vocal emotion recognition in patients with left (LCBL) or right (RCBL) cerebellar lesions, particularly for neutral or negative prosody, but RCBL made more misattributions than LCBL (Thomasson et al., 2021). Note that neuroimaging studies and meta-analyses have reported inconsistent results with bilateral cerebellar activation (Ceravolo et al., 2021; Imaizumi et al., 1997; Wildgruber et al., 2005), whereas others have found only left (Kotz et al., 2013) or right (Alba-Ferrara et al., 2011) activations during emotional prosody processing.

Although there is still much controversy, there is further evidence to support the involvement of the right BG and right cerebellar hemisphere in emotional prosody processing. However, neuroanatomical studies have suggested that there are cross-connections between these two structures. In nonhuman primates, it has been shown that the STN projects to the pontine nuclei and then to the contralateral cerebellar hemisphere (Bostan & Strick, 2010). Likewise in humans, fibers from the right/left STN extend contralaterally to the left/right cerebellum (Wang et al., 2020).

Regardless of lesion lateralization, there is a lack of evidence regarding the severity of the deficits depending on whether the BG or the cerebellum are affected. To the best of our knowledge, the only study to have compared patients with PD or cerebellar stroke (Adamaszek et al., 2019) on the recognition and discrimination of facial and auditory emotion reported greater impairment in patients with cerebellar lesions than in patients with PD, with more errors for all emotions, especially fear. This study lacked the statistical power needed to consider the lateralization variable in its analyses. Nevertheless, according to the above-mentioned studies, extremely heterogeneous patterns of deficits seem to be reported in patients in terms of both severity and nature, depending on the side of the lesions/degeneration. Thus, in the light of models describing a large and distributed network encompassing brain areas known to be involved in different stages of emotional prosody decoding (Grandjean, 2021; Péron et al., 2015; Schirmer & Kotz, 2006; Wildgruber et al., 2005), it would be interesting to investigate the differential roles of the BG and cerebellum in this process. Moreover, because these models describe specific right- and left-hemispheric involvement in the multiple stages of emotional prosody processing (Schirmer & Kotz, 2006), the hemispheric contributions of the BG/cerebellum need to be identified.

In this context, the purpose of this study was to explore the differential deficits brought about by BG degeneration versus cerebellar lesions in humans, considering the lateralization of the lesions (cerebellar stroke patients) or degeneration (PD). To this end, we first analyzed the vocal emotion recognition performances of 24 patients with PD, 24 patients with cerebellar stroke, and 24 HC, based on published data (Thomasson et al., 2021; Voruz et al., 2020). In the light of previous studies, we predicted that RCBL would be more impaired than LCBL, and LPD would display greater difficulties than RPD (Stirnimann et al., 2018;Thomasson et al., 2019 ; Voruz et al., 2020). Finally, and combining the two previous predictions, we expected to observe more deficits in RCBL than in LPD, and more deficits in LCBL than in RPD. Finally, we studied the impact of disease duration / time since stroke on patients’ emotional performances. To be valid, this type of comparative study must consider changes over time in the effects observed in the two populations. It is essential to address both the potential decline in performances with the progression of the neurodegenerative process in PD and the compensatory neural reorganization that follows cerebellar stroke (O'Halloran et al., 2012), to avoid misinterpreting the results. We therefore included disease duration / time since stroke as a variable of interest in our analyses. We predicted that the effects observed in our PD subgroups would be amplified over time, while the opposite pattern would be observed in our cerebellar stroke subgroups.

## Method

### Participants

We used datasets for 24 patients with PD and 24 patients with focal cerebellar lesions due to ischaemic stroke collected during previous studies (Thomasson et al., 2019; Voruz et al., 2020). The 24 patients with PD were all recruited at Rennes University Hospital (France) and were divided into two subgroups, based on the side of motor symptom onset: LPD (*n* = 12) and RPD (*n* = 12). This distinction was corroborated by an asymmetry index based on the lateralized items of Part III of the Unified Parkinson’s Disease Rating Scale (Fahn & Elton, 1987) (for details of this index, see Voruz et al., 2020). All patients with PD were tested *on levodopa*, meaning that they continued to receive their normal dopamine replacement therapy during testing. The two PD subgroups had comparable disease duration, age at motor symptom onset, cognitive functions, disease stage, motor functions (except for the asymmetry index), and dopamine replacement therapy (Table 2). The 24 patients with chronic cerebellar stroke were all recruited at Geneva University Hospitals and were divided into two subgroups: RCBL (*n* = 13), and LCBL (*n* = 11). These two subgroups were similar in terms of cerebellar ataxia, cognitive functions, and time since stroke (Table 3). All patients were French speakers. Exclusion criteria were 1) brainstem or occipital lesion (factor influencing clinical signs), 2) one or more other brain lesions, 3) diffuse and extensive white-matter disease, 4) other degenerative or inflammatory brain diseases, 5) confusion or dementia, 6) major psychiatric disease, 7) hearing aids, history of tinnitus or hearing impairment, as attested by the Montreal Toulouse auditory agnosia battery (PEGA) (Agniel et al., 1992) for stroke patients, and by a standard audiometric screening procedure (AT-II-B audiometric test) for the patients with PD, 8) younger than age 18 years, and 9) major language comprehension deficits precluding reliable testing. All tasks described below were designed to be highly feasible even for patients in clinical settings.

One matched HC group took part to this study, including 24 participants who had no history of neurological disorders, head trauma, anoxia, stroke, or major cognitive impairment, as attested by their score on the Mattis Dementia Rating Scale (MDRS) (Mattis, 1988) (range 139-144) or on the French version of the modified telephone interview for cognitive status (Lacoste & Trivalle, 2009) (range 32-43). They were all French speakers; none of them wore hearing aids or had a history of tinnitus or hearing impairment, as attested by their PEGA or AT-II-B scores.

The four patient subgroups (LPD, RPD, LCBL, and RCBL) and the HC group were matched for age (*p* = 0.86), handedness (*p* = 0.89), and sex (*p* = 0.97) (Table 1). Although there was no significant difference between the five groups on education level (*p* = 0.36), we preferred to add this variable to our statistical model to control for its potential effect on our data.

**Table 1:** Statistical results of clinical and healthy groups (LPD, RPD, LCBL, RCBL, and HC) comparisons on demographic and clinical data

	LPD (mean ± *SD*)	RPD (mean ± *SD*)	LCBL (mean ± *SD*)	RCBL (mean ± *SD*)	HC (mean ± *SD*)	Stat. value	*p* value
Age	58.75 ± 7.56	54.58 ± 6.87	62.37 ± 10.15	61.38 ± 12.33	60.37 ± 8.91	1.29	0.86
Education level	12.08 ± 3.45	12.17 ± 3.07	12.64 ± 4.10	16.00 ± 4.67	13.58 ± 2.57	8.55	0.36
Sex	1.42 ± 0.51	1.42 ± 0.51	1.54 ± 0.52	1.38 ± 0.51	1.46 ± 0.51	0.73	0.97
Handedness	1.17 ± 0.39	1.00 ± 0.00	1.09 ± 0.30	1.08 ± 0.28	1.08 ± 0.28	2.17	0.89
Disease duration / Time since stroke (days)	4227.92 ± 1686.76	4197.50 ± 1439.11	1301.45 ± 1351.48	517.08 ± 430.34	NA	31.99	0.03*

*HC* healthy controls, *LCBL* patients with left cerebellar stroke, *LPD* patients with Parkinson’s disease exhibiting predominantly left-sided motor symptoms, *RCBL* patients with right cerebellar stroke, *RPD* patients with Parkinson’s disease exhibiting predominantly right-sided motor symptoms, *SD* standard deviation

*p < 0.05

### Procedure

#### Motor assessment

Patients with PD were scored on the UPDRS-III, Hoehn and Yahr scale (Hoehn & Yahr, 1998) and Schwab and England scale (Schwab, 1969), whereas patients with cerebellar stroke were scored on the Scale for the Assessment and Rating of Ataxia (Schmitz-Hubsch et al., 2006).

#### Neuropsychological and psychiatric assessment

First, a global cognitive scale was administered to each group of patients: the MDRS (Mattis, 1988) for patients with PD, and the Montreal Cognitive Assessment (Nasreddine & Patel, 2016) for patients with cerebellar stroke. This was followed by a series of tests to assess frontal executive functions: Categorical and Literal Fluency Test (Cardebat et al., 1990) and Action (Verb) Fluency Task (Woods et al., 2005) for both groups of patients; Trail Making Test (TMT) (Reitan, 1958) and Stroop test (Stroop, 1935) for patients with PD; and Frontal Assessment Battery ((Dubois et al., 2000) for patients with cerebellar stroke. Finally, psychiatric symptoms were investigated, using the State-Trait Anxiety Inventory (Spielberger et al., 1983) to assess anxiety in patients with PD and the Toronto Alexithymia Scale (Bagby et al., 1994) to assess alexithymia in patients with cerebellar stroke. Because depressive symptoms are observed in both pathologies, we administered the Montgomery-Åsberg Depression Rating Scale ((Montgomery & Åsberg, 1977) to the patients with PD and the Beck Depression Inventory (Steer et al., 2001) to the patients with cerebellar stroke. Finally, the Apathy Evaluation Scale (AES) (Marin et al., 1991) was used to assess the potential presence of apathy symptoms in the two patient groups.

#### Vocal emotion recognition task

We administered a validated emotional prosody recognition task that had already been used in PD (Péron et al., 2017) and cerebellar stroke (Thomasson et al., 2019). In this task, participants listen to meaningless speech (60 pseudowords) expressed in five different emotional prosodies (anger, fear, happiness, neutral, and sadness). For each pseudoword, they have to indicate the extent to which it expresses different emotions, by moving a cursor along a continuous analog scale (emotion scales display: happiness, anger, fear, and sadness, neutral, and surprise) ranging from “No emotion expressed” to “Emotion expressed with exceptional intensity.” For a detailed description of the task, see Thomasson et al. (2019).

### Statistical analysis

#### Clinical, demographic, and neuropsychological data

Because this data did not follow a normal distribution (Shapiro–Wilk test, all *p* < 0.05), comparisons between the five groups (LPD, RPD, LCBL, RCL, and HC) were performed by using Kruskal-Wallis tests. Other comparisons (LPD vs. RPD or LCBL vs. RCBL) were performed using Mann-Whitney tests for two independent groups. However, the data of the variable “*Age*” were normally distributed so we performed single-factor analysis of variance (ANOVA). If the ANOVA test yielded a significant difference, pairwise *t*-tests for two independent groups were performed to determine which groups differed from one another. We performed FDR correction for multiple comparisons.

#### Vocal emotion recognition data

A *frequentist* general linear mixed model (GLMM) approach was adopted to compare the performances of the five groups (HC, LPD, RPD, LCBL, and RCBL). Five groups were created to avoid an unbalanced design where the two patient groups (LPD vs. CBL) and lesion side (left vs. right) were included as between-participants variables. Moreover, because the data distribution was characterized by an excess of 0 and an overdispersion of the data, we ran a GLMM with a compound Poisson-Tweedie distribution (best fit to the data reflected by lowest Akaike information criterion, AIC) using the GLMMTMB package (Brooks et al., 2017). The model with the Tweedie distribution presented a better fit of the data than the models with a Gaussian distribution (Akaike information criterion: 139783.6 (Tweedie); 239415.2 (Gaussian)). It allowed us to control for random effects such as interindividual variability, in addition to fixed effects. This GLMM was performed with emotion (5 levels) and scale (6 levels) as within-participants factors, group (HC, LPD, RPD, LCBL, and RCBL) as the between-participants variable, and participant as the random factor.

For each model, we ran contrasts between the groups for each prosodic category and each scale, based on the GLMM model using the phia package in R software (version 2.15.0). The *p* value yielded by the contrasts was false discovery rate (FDR) corrected (threshold of *p* = 0.05). However, as Tweedie analysis can be oversensitive to spurious effects, we performed a control analysis using the BayesFactor package. Bayesian *t* tests were performed between the groups, and significant Tweedie effects were selected if the Bayes factor exceeded 3 (for details, see Benis et al., 2020).

We addressed the potential confound of time by including disease duration / time since stroke as a covariate in our analyses of emotion processing**.** To simplify our statistical model, we decided to merge the scale and emotion variables into one (named *effect*). Moreover, to reduce the overdispersion of the data, we used a Log10 transformation for the disease duration / time since stroke variable. We therefore calculated another GLMM model, including the disease duration / time since stroke variable as a covariate of interest in a three-way interaction with group and effect variables. This interaction would be a marker of differing courses of the deficits observed in the two patient groups. We then performed contrasts and linear trend analyses using the emmeans package (Lenth, 2019) to study predictions of the emotional performances of the four patient subgroups at different points in the disease duration / time since stroke.

Finally, we looked for correlations between the clinical and emotional data for the patient groups using Spearman’s rank test, as the distribution of the data was not normal. To avoid Type-I errors, we only included emotional variables that differed significantly across the four patient subgroups in the analyses.

## Results

### Clinical, demographic, and neuropsychological data (Tables 1, 2, and 3)

#### Intrapathology comparisons

Concerning patients with PD, we found trend differences between LPD and RPD on the motor asymmetry index (Table 2). Comparisons between the two patient subgroups with cerebellar stroke (LCBL and RCBL) failed to reveal any significant differences (Table 3).

**Table 2:** Statistical results of comparisons between the two subgroups of patients with PD (LPD and RPD) on motor, neuropsychological and psychiatric data

	LPD (mean ± *SD*)	RPD (mean ± *SD*)	Stat. value	*p* value
L-DOPA equivalent dose (mg/day)	1302.92 ± 598.45	1295.58 ± 582.22	0.20	0.96
Hoehn and Yahr score on dopa	1.04 ± 0.86	9.46 ± 28.84	0.58	0.86
Hoehn and Yahr score off dopa	2.12 ± 0.74	10.92 ± 28.38	1.64	0.44
Schwab and England score on dopa	90.00 ± 8.53	83.50 ± 27.19	0.09	0.97
Schwab and England score off dopa	72.50 ± 11.38	58.62 ± 33.52	-0.87	0.86
UPDRS-III motor score on dopa	8.37 ± 6.66	6.96 ± 4.72	-0.32	0.89
UPDRS-III motor score off dopa	30.87 ± 9.37	34.04 ± 16.09	0.32	0.89
Asymmetry index	-2.67 ± 2.31	0.50 ± 1.19	3.23	0.07
MDRS (total score)	140.67 ± 1.72	139.83 ± 3.21	-0.58	0.86
Categorical verbal fluency	27.25 ± 13.79	30.33 ± 10.67	0.84	0.85
Action (Verb) Fluency	14.50 ± 6.87	14.00 ± 6.69	-0.06	0.97
Phonemic verbal fluency	20.83 ± 7.75	22.00 ± 5.62	0.66	0.85
Stroop Test - Interference	11.90 ± 28.77	2.90 ± 6.88	-0.34	0.89
TMT B-A	68.42 ± 81.38	64.75 ± 41.85	0.84	0.85
AES	29.25 ± 7.24	32.00 ± 5.95	0.72	0.85
MADRS	2.92 ± 3.53	13.92 ± 27.85	1.82	0.36
STAI-A State	35.17 ± 21.31	45.75 ± 21.17	2.26	0.21
STAI-B Trait	40.83 ± 21.51	48.92 ± 18.76	1.97	0.33

*AES* Apathy Evaluation Scale, *L-DOPA* levodopa-equivalent daily dose, *LPD* patients with Parkinson’s disease exhibiting predominantly left-sided motor symptoms, *MADRS* Montgomery-Åsberg Depression Rating Scale, *MDRS* Mattis Dementia Rating Scale, *RPD* patients with Parkinson’s disease exhibiting predominantly right-sided motor symptoms, *SD* standard deviation, *STAI* State-Trait Anxiety Inventory, *TMT* Trail Making Test, *UPDRS* Unified Parkinson’s Disease Rating Scale

*p < 0.05

**Table 3:** Statistical results of comparisons between the two subgroups of patients with cerebellar stroke (LCBL and RCBL) on motor, neuropsychological and psychiatric data

	LCBL (mean ± *SD*)	RCBL (mean ± *SD*)	Stat. value	*p* value
SARA	2.20 ± 2.76	1.70 ± 2.26	-0.57	0.85
MOCA (total score)	24.82 ± 3.74	23.92 ± 2.90	-0.55	0.85
Categorical verbal fluency	18.18 ± 7.52	17.31 ± 7.72	0.35	0.89
Action (Verb) Fluency	14.45 ± 4.32	13.08 ± 8.28	0.69	0.85
FAB	14.20 ± 2.48	15.50 ± 2.50	1.17	0.83
AES	5.70 ± 6.50	1.60 ± 3.06	1.17	0.83
BDI-II	11.82 ± 6.66	13.50 ± 6.81	0.61	0.85
TAS-20	54.20 ± 17.66	53.11 ± 16.42	0.04	0.98

*AES* Apathy Evaluation Scale, *BDI-II* Beck Depression Inventory, *FAB* Frontal Assessment Battery, *LCBL* patients with left cerebellar stroke, *MOCA* Montreal Cognitive Assessment, *RCBL* patients with right cerebellar stroke, *SARA* Scale for the Assessment and Rating of Ataxia, *SD* standard deviation, *TAS-20* Toronto Alexithymia Scale

#### Interpathology comparisons

We found a significant difference between the four patient subgroups on disease duration / time since stroke. There were more months between the date of disease onset and the date of testing in LPD than in either LCBL (*z* = 3.55, *p* < 0.001) or RCBL (*z* = 4.22, *p* < 0.001). More months between the date of disease onset and the date of testing also were observed in RPD compared with LCBL (*z* = 3.41, *p* < 0.001) and RCBL (*z* = 4.21, *p* < 0.001) (Table 1). In addition, a significant difference was found on the categorical verbal fluency score, as RPD scored higher on this task than RCBL (*z* = 2.80, *p* < 0.01). For the AES score, a significant difference also was observed, with RPD presenting more apathy symptoms than either LCBL (*z* = 3.93, *p* < 0.001) or RCBL (*z* = 3.95, *p* < 0.001). Moreover, LPD manifested more apathy symptoms than either LCBL (*z* = 3.79, *p* < 0.01) or RCBL (*z* = 3.95, *p* < 0.01).

### Vocal emotion recognition task

#### First level of analysis: group effects

GLMM analysis revealed a significant Group × Emotion × Scale three-way interaction, showing that group (HC, LPD, RPD, LCBL, RCL) influenced the recognition of emotional prosody, χ^2^(80) = 208.14, *p* < 0.0001.

Other main and interaction effects were as follows: group: χ2(4) = 4.85, *p* = 0.30; emotion: χ^2^(4) = 37.44, *p* < 0.0001; scale: χ^2^(5) = 107.7, *p* < 0.0001; Group × Emotion: χ^2^(16) = 3.30, *p* = 0.99; Group × Scale: χ^2^(20) = 63.64, *p* < 0.0001; and Emotion × Scale: χ^2^(20) = 5768.6, *p* < 0.0001.

Contrasts for each vocal emotion and each rating scale were performed, with FDR-corrected *p* values, and controlled by Bayesian *t*-test analysis. All the effects are summarized in Table 4.

**Table 4:** Significant contrasts between each group (LPD, RPD, LCBL, RCBL, HC) on each vocal emotion and each rating scale, revealed by the second level of analysis

Emotion	Scale	Contrast direction	Stat. value	*p* value	Bayes factor
Anger	Fear	LCBL > HC	-2.27	0.05	5.38
	Surprise	RCBL > RPD	2.32	0.04	9.16
	Sadness	LPD > RPD	-4.71	<0.001	7,372.80
		RCBL > RPD	3.53	<0.001	5.62
		LCBL > RPD	4.43	<0.001	12.28
		HC > RPD	3.53	<0.001	3.54
Happiness	Surprise	LPD > RPD	-2.37	0.03	110.77
		LPD > LCBL	-2.25	0.05	71.95
	Sadness	LPD > RPD	-2.52	0.02	25.59
		HC > RPD	2.89	<0.01	20.54
Neutral	Anger	LPD > RPD	-7.50	<0.001	4,846.63
		LPD > LCBL	-2.56	0.02	35.38
		LPD > RCBL	-2.56	0.02	9.86
		LPD > HC	-5.32	<0.001	208,811.68
		LCBL > RPD	5.04	<0.001	3.08
		RCBL > RPD	5.24	<0.001	3.95
	Happiness	RCBL > LCBL	2.53	0.02	4.45
		RCBL > HC	-2.28	0.04	20.61
	Fear	LPD > RPD	-3.14	<0.01	247.86
		LPD > HC	-3.16	<0.01	5,316.21
		RCBL > HC	-2.67	0.01	3.29
	Sadness	LPD > RPD	-3.45	<0.001	9,122.35
		LCBL > RPD	4.37	<0.001	73.99
Fear	Happiness	RCBL > LCBL	4.64	<0.001	5.91
	Surprise	RCBL > RPD	3.23	0.003	55,663.05
Sadness	Anger	LPD > RPD	-2.88	0.008	27.27
		LPD > HC	-2.97	0.006	1,589.45
	Surprise	RCBL > RPD	3.30	0.002	3.08
		RCBL > HC	-3.43	0.001	20.43

*HC* healthy controls, *LCBL* patients with left cerebellar stroke, *LPD* patients with Parkinson’s disease exhibiting predominantly left-sided motor symptoms, *RCBL* patients with right cerebellar stroke, *RPD* patients with Parkinson’s disease exhibiting predominantly right-sided motor symptoms

##### Intrapathology comparisons

Among cerebellar stroke patients, RCBL made more emotional misattributions (higher ratings on Happiness scale when listening to neutral or fearful prosody) than LCBL. Among patients with PD, LPD made more emotional misattributions (higher ratings on Sadness scale when listening to neutral, happy or angry prosody; higher ratings on Anger scale when listening to neutral or sad prosody; higher ratings on Fear scale when listening to neutral prosody; higher ratings on Surprise scale when listening to happy prosody) than RPD.

##### Interpathology comparisons

We observed more vocal emotional recognition deficits (higher ratings on Sadness scale when listening to angry or neutral prosody; higher ratings on Anger scale when listening to neutral prosody) in LCBL than in RPD. Interestingly, we also noticed that RCBL performed more poorly than RPD (higher ratings on Surprise scale when listening to angry, fearful or sad prosody; higher ratings on Sadness scale when listening to anger prosody; higher ratings on Anger scale when listening to neutral prosody). Moreover, compared with LCBL, LPD attributed significantly higher Surprise ratings to happiness prosody, and higher Anger ratings to neutral prosody. Finally, results showed that, compared with RCBL, LPD attributed significantly higher Anger ratings to neutral prosody.

#### Second level of analysis: Effect of disease duration / time since stroke on vocal emotion decoding

A second GLMM was performed with the variables group, effect (pooled variables scale and emotion) and disease duration / time since stroke (Log 10-transformed). Results revealed a significant Group × Effect × Disease duration / Time since stroke three-way interaction, χ^2^(87) = 225.82, *p* < 0.0001. Contrasts were then performed, with FDR-corrected *p* values. Only significant contrasts that were already significant in the first GLMM model (Table 3) are reported here (Table S1).

##### Anger

**Fig. 1:**
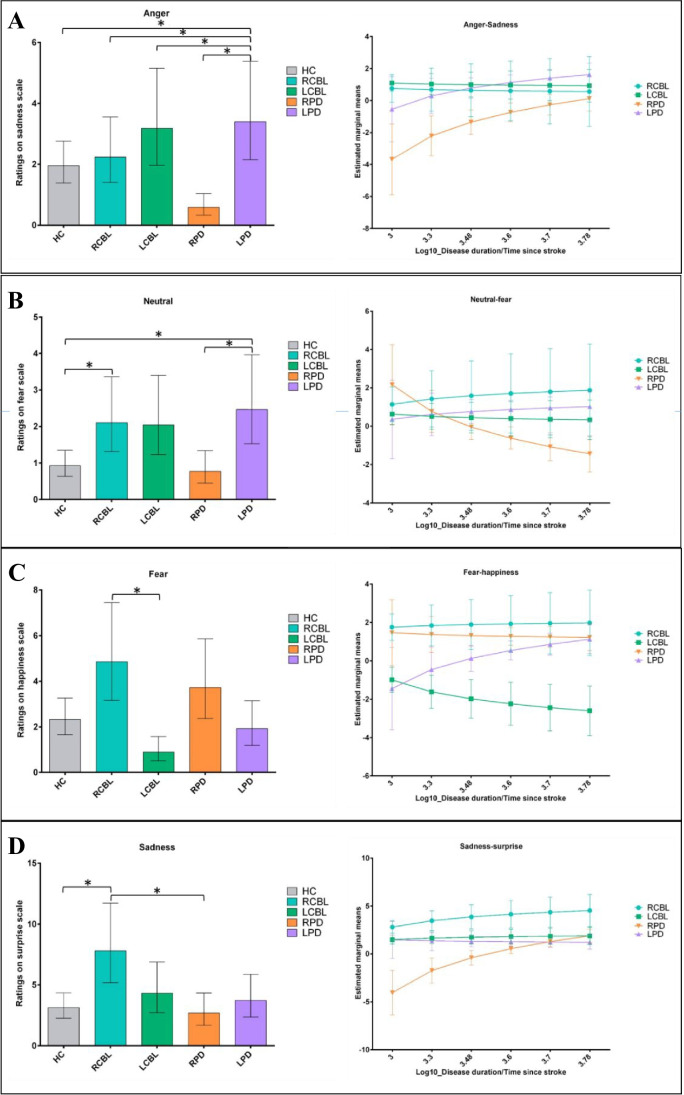
**a** Mean ratings on the Sadness scale (left) and differential impact of disease duration / time since stroke (right) when the stimulus was anger for the HC (grey), RCBL (blue), LBCL (green), RPD (orange), and LPD (purple) groups. **b** Mean ratings on the Fear scale (left) and differential impact of disease duration / time since stroke (right) when the stimulus was neutral for the HC (grey), RCBL (blue), LBCL (green), RPD (orange), and LPD (purple) groups. **c** Mean ratings on the Happiness scale (left) and differential impact of disease duration / time since stroke (right) when the stimulus was fear for the HC (grey), RCBL (blue), LBCL (green), RPD (orange), and LPD (purple) groups. **d** Mean ratings on the Surprise scale (left) and differential impact of disease duration / time since stroke (right) when the stimulus was sadness for the HC (grey), RCBL (blue), LBCL (green), RPD (orange), and LPD (purple) groups. *p < 0.05

Angry stimuli on the Sadness scale (Fig. 1a): significant differences were observed between RPD and both subgroups of cerebellar stroke patients (RPD-RCBL; difference between the two trends, *t*(17037) = −9.04, *p* = 0.03; RPD-LCBL: difference between the two trends, *t*(17037) = −8.68, *p* = 0.02). More specifically, a significant positive trend was observed for RPD, *t*(17037) = −3.71, *p* < 0.001, reflecting an increase in their emotional misattributions over time, compared with RCBL and LCBL.

##### Neutral

Neutral stimuli on the Fear scale (Fig. 1b): a significant difference was observed between RPD and RCBL (difference between the two trends: *t*(17037) = 2.65, *p* = 0.02). A significant negative trend was observed for RPD, *t*(17037) = −2.55, *p* = 0.03, reflecting a decrease in their emotional misattributions over time, compared with RCBL.

##### Fear

Fear stimuli on the Happiness scale (Fig. 1c): significant differences were observed between LCBL and RCBL (difference between the two trends: *t*(17037) = 2.58, *p* = 0.03) and between LCBL and LPD (difference between the two trends: *t*(17037) = −2.96, *p* = 0.01). More specifically, a significant negative trend was observed for the LCBL subgroup, *t*(17037) = −3.71, *p* < 0.001, reflecting a decrease in their emotional misattributions over time, compared with RCBL and LPD.

##### Sadness

Sadness stimuli on the Surprise scale (Fig. 1d): significant differences were observed between RPD and both cerebellar stroke subgroups (RPD-RCBL: difference between the two trends, *t*(17037) = −2.76, *p* = 0.02; RPD-LCBL: difference between the two trends, *t*(17037) = −3.80, *p* < 0.001), as well as the LPD subgroup (RPD-LPD: difference between the two trends, *t*(17037) = −3.26, *p* < 0.001). More specifically, a significant positive trend was observed for the RPD subgroup, *t*(17037) = 4.19, *p* < 0.001, reflecting an increase in their emotional misattributions over time, compared with the RCBL, LCBL, and LPD subgroups.

### Relationship between vocal emotion decoding and secondary variables

Spearman’s rank tests revealed a significant correlation between ratings on the Sadness scale for neutral prosody and scores on the categorical fluency task (*r* = −0.36, *p* = 0.01). We also observed further significant correlations between emotional variables (ratings on the Surprise and Sadness scales for angry prosody, ratings on the Sadness scale for happy prosody, and ratings on the Fear scale for neutral prosody) and the AES score (respectively, *r* = −0.46, *p* = 0.002; *r* = −0.31, *p* = 0.04; *r* = −0.32, *p* = 0.03; *r* = −0.33, *p* = 0.02). We added the categorical verbal fluency or AES variables to our GLMM model, taking relevant factors and interactions of interest into account, to see if one of these variables explained a significant part of the variance of our results. We calculated the AIC and Bayesian information criterion (BIC) to see whether the models containing the categorical fluency score or AES score variables presented a better fit of the data than the model that did not contain them. The lower the AIC or BIC value, the better the fit. For the model containing no additional variable, AIC was 86571.2 and BIC was 86624.9. When the categorical fluency scores were included in the model, they did not significantly affect emotional ratings (χ^2^ = 0.25, *p* = 0.62, AIC = 86572.9, BIC = 86634.3). Therefore, participants’ lack of verbal self-activation in the categorical fluency task did not explain their judgments in the emotional prosody recognition task. By contrast, when we included AES scores, results showed that this variable significantly affected emotional ratings (χ^2^ = 5.79, *p* = 0.02, AIC = 86567.4, BIC = 86628.8).

## Discussion

The first aim of this retrospective study was to compare the performances of patients with PD or cerebellar stroke on vocal emotion recognition, in order to explore the differential roles of the cerebellum and BG in emotional prosody decoding, taking hemispheric dysfunction into account. More specifically, by studying patterns of deficits observed in patients with PD (basal ganglia dysfunction) and patients with cerebellar stroke, we were able to directly ascertain whether lateralized damage to one of these two sets of structures caused similar or completely different emotional deficits. Accordingly, we administered a validated and sensitive emotional prosody recognition task that allowed us to quantify misattributions. We analysed the emotional data we collected by comparing the performances of four patient subgroups (RPD, LPD, RCBL, and LCBL) and one matched HC group.

### Intrapathology comparisons

Concerning cerebellar stroke patients, those with right-hemispheric lesions made more emotional misattributions (specifically when they listened to neutral or negative prosody) than patients with left lesions. Cerebellar involvement in the processing of negative emotions has been mentioned several times in the literature (Adamaszek et al., 2014; Adamaszek et al., 2017; Baumann & Mattingley, 2012; Ferrucci et al., 2012; Paradiso et al., 1999; Schutter & van Honk, 2009; Thomasson et al., 2019), and it has been suggested that a complex cortico-cerebellar network is specific to aversive stimuli (Moulton et al., 2011). By contrast, Adamaszek et al. (2019) found that patients with right cerebellar lesions tended to have better total scores on the Tübingen affect battery than patients with left cerebellar lesions. However, major methodological differences between this study and ours can probably explain the divergent results (battery assessing facial and vocal emotions vs. emotional prosody only, categorization task vs. task using continuous scales, presence of semantic content in stimuli vs. use of nonwords, sample size). Concerning patients with PD, only left-lateralized patients exhibited a vocal emotion deficit. Stirnimann et al. (2018), who observed the same results, suggested that a right orbitofrontal-BG coupling is specifically involved in the vocal emotion recognition deficit observed in PD. Interestingly, some authors (Voruz et al., 2020) have postulated that the emotional deficits exhibited by LPD are normalized by STN DBS through the functional resynchronization of the limbic loop, thereby restoring the cerebral and cerebellar interactions needed for intact vocal emotion processing.

### Interpathology comparisons

Results showed that RPD performed better than all the other patient subgroups (LPD, RCBL, and LCBL), confirming our prediction that LCBL would display more deficits than RPD. In addition, LPD made more misattributions than LCBL, as they rated happy prosody significantly higher on the Surprise scale, and neutral prosody significantly higher on the Anger scale. Thus, taken together with the intragroup results, it would appear that LPD (i.e., with greater right hemispheric brain dysfunction) and RCBL were the subgroups with the most pronounced deficit for emotion vocal recognition. These results are in line with findings concerning the involvement of the right cerebellum (Thomasson et al., 2019; Thomasson et al., 2021) and right BG (Garrido-Vásquez et al., 2013; Stirnimann et al., 2018; Ventura et al., 2012) in vocal emotion decoding. However, our prediction that RCBL would have greater difficulty than LPD could not be confirmed, as we only observed one significant difference between these two subgroups, and it went in the opposite direction (LPD rated neutral prosody significantly higher on the Anger scale than RCBL did). Nevertheless, the nature of the errors made by the different patient subgroups could be an interesting factor to take into account. For example, the misattributions made by RCBL seemed to reflect a deficit in processing the valence of the stimuli. According to the component process model of emotions, during the intrinsic pleasantness check (step that occurs at a very low level of processing at first stage), the brain assesses whether a stimulus event is likely to result in pleasure or in displeasure, based on a given feature of the stimulus (Sander et al., 2005). In the light of this theory, we can assume that this stimulus evaluation check is disturbed following a right cerebellar stroke. Furthermore, misattributions by LPD (i.e., patients with greater right hemispheric brain dysfunction) were similar to the error patterns (i.e., errors between emotional prosodies of the same valence) that can be observed in our patients group with left cerebellar lesions. Thus, these patients can successfully perform the intrinsic pleasantness check but are impaired at a higher level of processing. These findings are relevant regarding the neuro-anatomical cross-connectivity between the BG (more specifically the STN) and the cerebellum (Wang et al., 2020). Animal and human studies have shown that both the STN and the cerebellum are involved in high-level evaluative judgments (Deverett et al., 2018; Roldan Gerschcovich et al., 2011; Voon et al., 2017). More specifically, some authors (Wang et al., 2020) have suggested that a specific pathway, linking up the regions of the medial prefrontal cortex/mesial Brodmann area 8, right STN and left Crus I, is elicited during high-level conscious processes. We therefore suggest that hemispheric specialization of the cerebellum and BG depends on the level of emotional stimulus processing (Grandjean & Scherer, 2008; Leventhal & Scherer, 1987). Disturbances in the left cerebellum and right STN (LPD) appear to lead to deficits in higher cognitive processes involved in a later stage of emotional prosody decoding, whereas lesions in the right cerebellar hemisphere appear to induce difficulties in the earlier stages (deficits in intrinsic pleasantness check or in feature processing required for this evaluation check). However, our results did not reveal an error pattern similar to RCBL in RPD. The hemispheric specialization hypothesis, which distinguishes between early and late emotion processing, has yet to be debated in the literature. Concerning BG, some authors have suggested that the right BG are involved in the early processing stage (Garrido-Vásquez et al., 2013), and the left BG subtend the late stage of emotional prosody processing (Paulmann et al., 2011), whereas others have postulated that the right and left STN are involved in both early and late stages (Péron et al., 2017).

### Limitations

One limitation of the current study was the small sample size, which we tried to overcome by applying strict corrections of *p* values in our statistical analyses and considering interindividual differences. The present study yielded several interesting and reproducible positive results. While we cannot exclude the possibility that some additional positive results might have emerged with a larger sample size, the present results already paint a very interesting portrait of the similarities and differences between the two populations, in terms of emotional prosody deficits. Moreover, the Bayesian data analysis allowed us to disentangle negative results attributable to insufficient power (BF [0.33 3]) from *true* negative results emerging from the validation of H0 (BF < 1/3). This additional information allowed us to discuss negative results in the light of this difference, whilst limiting the bias caused by our small sample size. Nevertheless, further studies with larger samples are needed to confirm our results. Another limitation was the dopamine replacement therapy of patients with PD, as this may enhance compensatory mechanisms. Even if studies investigating emotional prosody recognition in PD have not provided convincing evidence that dopamine replacement therapy enhances emotional performances (Perón et al., 2012), it would be useful to conduct another study with on and off dopa conditions. Additionally, as this study had another major limitation, namely the comparison between two populations with totally different neurological conditions (i.e., neurodegenerative pathology vs. stroke with potential partial recovery), a second aim was to investigate whether disease duration / time since stroke had a differential impact on emotional prosody recognition. Results revealed that unlike the two cerebellar stroke subgroups, RPD made increasing numbers of emotional misattributions (for angry and sad prosody) over time. Interestingly, the literature suggests that RPD undergo a more substantial cognitive decline, owing to a greater reduction in white matter integrity (Pelizzari et al., 2020) or damage in the left cortical hemisphere (Claassen et al., 2016). Interestingly, analyses also revealed a trend toward a difference between RPD and LPD on the motor asymmetry index, reflecting a greater tendency toward bilateral motor symptoms in RPD. These results also raise questions about the role of the cerebellum in PD. At the beginning of the disease, the cerebellum may compensate for deficient BG function, but this compensation presumably decreases as new symptoms emerge and become more severe (Wu & Hallett, 2013). Thus, in the light of neuro-anatomical cross-connectivity between the BG (more specifically the STN) and the cerebellum, we can assume that at the beginning of the disease, the relatively well-preserved performances of RPD for the judgment of emotions conveyed by the voice is due to efficient compensation by the right cerebellar hemisphere. The question of right cerebellar functional specialization for emotional prosody processing takes on even more meaning when we consider the improvement in LCBL performances over time, in contrast to those of RCBL and LPD, for rating fear prosody on the Happiness scale. Thus, when the right cerebellar hemisphere is affected, whether by an acute event (stroke) or via a neurodegenerative process as a result of right hemispheric brain dysfunction (PD), a persistence or increase in difficulties in emotional prosody processing may be observed. Consequently, more studies are needed to investigate the functional hemispheric specialization of the cerebellum, and its potential functional role in the compensation of the emotional effects related to neurodegenerative pathologies such as PD. Studies have reported abnormal cerebellar functional connectivity in cognitively impaired patients with PD (Kawabata et al., 2018; Maiti et al., 2020) or depression (Wang et al., 2018). This suggests that functional brain rehabilitation therapies targeting the cerebellum could be considered for patients with PD. Promising effects have already been demonstrated in patients with PD undergoing cerebellar transcranial direct current stimulation (tDCS). An improvement in the recognition of sad facial expressions was demonstrated in nine patients receiving anodal tDCS applied for 5 consecutive days over the cerebellum (Ruggiero et al., 2021). This empirical study therefore allowed to identify the neural mechanisms underlying the emotion recognition in facial modality and consequently to extends current knowledge on the important role of the cerebellum in emotional information processing. Overall, by providing more information about the roles of the affected and unaffected hemispheres and the severity of patients’ emotional impairment, whilst considering changes in emotional disorders over time, the present study clearly highlights the clinical issues. There may be a window of opportunity for early interventions aimed at promoting compensation strategies based on the capacities of the preserved hemisphere, thereby limiting the impact on patients’ everyday lives.

Taken together, the results of this innovative study confirm BG and cerebellar involvement in emotional prosody processing. They revealed differential error patterns according to the lateralization of the hemispheric lesion/brain degeneration. This points to a crossed hemispheric specialization between these two structures, with differential roles during both the early stages (sensory processing) and the later stages (appraisal) of emotional prosody processing. Moreover, by considering disease courses over time, this study highlighted different patterns of progression, according to the lateralization of the lesion/brain degeneration, thus reinforcing the hypothesis that the cerebellum plays a major role in PD. Nevertheless, future studies will need to investigate the differential functional roles played by the cerebellum and BG in emotional processing. These results should be seen in the light of the complexity of neuro-anatomical connections described in the literature, especially cortico-subcortical connections (e.g., between the cerebellum and cerebral cortex) and the ones between the BG and cerebellum. These pathways are not always exclusively contralateral, as they can sometimes also be ipsilateral (Karavasilis et al., 2019; Wang et al., 2020).

## Conclusions

The present study tested-for the first time to our knowledge-how hemispheric damage/brain degeneration in patients with cerebellar stroke/PD impairs the recognition of emotional prosody. When we considered the lateralization of the impairment, we observed more misattributions by both RCBL and LPD. These results highlighted the specific involvement of the cerebellum in vocal emotional decoding, but also the key role of the right BG, probably in higher-level emotional processing. Additional analyses considering disease duration / time since stroke showed a worsening of RPD’s performances over time. Further studies are needed to better understand the functional interplay between these two structures, as well as the timing of their differential roles during the different stages of limbic (but also motor and associative) processing. A more systematic consideration of the lateralization of impairment might confirm the crossed functional specialization between the BG and cerebellum suggested by our study of error patterns, which could have a major impact on the clinical management and potential rehabilitation strategies for the patients.

## Supplementary Information

ESM 1 (DOCX 220 kb)

## Abbreviations

ANOVA: Analysis of variance
FDR: False discovery rate
fMRI: Functional magnetic resonance imaging
GLMM: Generalized linear mixed model
PD: Parkinson’s disease
LPD: Patients with Parkinson’s disease exhibiting predominantly left-sided motor symptoms
RPD: Patients with Parkinson’s disease exhibiting predominantly right-sided motor symptoms
RCBL: Patients with right cerebellar lesions
LCBL: Patients with left cerebellar lesions
STN: Subthalamic nucleus
DBS: Deep brain stimulation
VLSM: voxel-based lesion-symptom mapping
PEGA: Montreal Toulouse auditory agnosia battery
MDRS: Mattis Dementia Rating Scale
UPDRS-III: Unified Parkinson’s Disease Rating Scale Part III
AES: Apathy Evaluation Scale
AIC: Akaike information criterion
BIC: Bayesian information criterion
TMT: Trail-Making Test
