# Supplementary material for: Crossed functional specialization between the basal ganglia and cerebellum during vocal emotion decoding: Insights from stroke and Parkinson’s disease
Source: Cogn Affect Behav Neurosci. 2022 Apr 26;22(5):1030–43. doi: 10.3758/s13415-022-01000-4 (PMC9458588; doi:10.3758/s13415-022-01000-4)
Supplement: Supplementary file 1 — (DOCX 220 kb) [file 13415_2022_1000_MOESM1_ESM.docx]

**Supplementary** **material**

**Table S1. Significant trends and contrasts between groups (LPD, RPD, LCBL, RCBL).**

| Emotion | Scale | RPD | LPD | RCBL | LCBL | Group comparison | *t* ratio | *p* |
| --- | --- | --- | --- | --- | --- | --- | --- | --- |
| **Anger** | Sadness | *t* = 2.78; ***p* = 0.02*** | *t* = 1.70; *p* = 0.21 | *t* = -0.27; *p* = 0.90 | *t* = -0.44; *p* = 0.83 | RCBL-LCBL | -2.09 | 0.99 |
|  |  |  |  |  |  | RCBL-LPD | -6.73 | 0.24 |
|  |  |  |  |  |  | RCBL-RPD | -9.04 | **0.03*** |
|  |  |  |  |  |  | LCBL-LPD | -6.36 | 0.19 |
|  |  |  |  |  |  | LCBL-RPD | -8.68 | **0.02*** |
|  |  |  |  |  |  | LPD-RPD | -6.83 | 0.61 |
| **Neutral** | Fear | *t* = -2.55; ***p* = 0.03*** | *t* = 0.5; *p* = 0.81 | *t* = 0.89; *p* = 0.60 | *t* = -0.78; *p* = 0.66 | RCBL-LCBL | 1.14 | 0.46 |
|  |  |  |  |  |  | RCBL-LPD | 0.05 | 0.98 |
|  |  |  |  |  |  | RCBL-RPD | 2.65 | **0.02*** |
|  |  |  |  |  |  | LCBL-LPD | -0.70 | 0.70 |
|  |  |  |  |  |  | LCBL-RPD | 2.26 | 0.07 |
|  |  |  |  |  |  | LPD-RPD | 2.21 | 0.08 |
| **Fear** | Happiness | *t* = -0.22; *p* = 0.92 | *t* = 1.91; *p* = 0.14 | *t* = 0.40; *p* = 0.85 | *t* = -3.71; ***p* < .001*** | RCBL-LCBL | 2.58 | **0.03*** |
|  |  |  |  |  |  | RCBL-LPD | -1.61 | 0.24 |
|  |  |  |  |  |  | RCBL-RPD | 0.38 | 0.86 |
|  |  |  |  |  |  | LCBL-LPD | -2.96 | **0.01*** |
|  |  |  |  |  |  | LCBL-RPD | -1.15 | 0.45 |
|  |  |  |  |  |  | LPD-RPD | 1.62 | 0.23 |
| **Sadness** | Surprise | *t* = 4.19; ***p* < .001*** | *t* = -0.19; *p* = 0.93 | *t* = 3.04; *p* < .01* | *t* = 1.14; *p* = 0.46 | RCBL-LCBL | 1.99 | 0.12 |
|  |  |  |  |  |  | RCBL-LPD | 1.43 | 0.32 |
|  |  |  |  |  |  | RCBL-RPD | -2.76 | **0.02*** |
|  |  |  |  |  |  | LCBL-LPD | 0.49 | 0.81 |
|  |  |  |  |  |  | LCBL-RPD | -3.80 | **< .001*** |
|  |  |  |  |  |  | LPD-RPD | -3.26 | **< .001*** |

*Note.* LPD: patients with Parkinson’s disease exhibiting predominantly left-sided motor symptoms; RPD: patients with Parkinson's disease exhibiting predominantly right-sided motor symptoms; LCBL: patients with left cerebellar stroke; RCBL: patients with right cerebellar stroke. * *p* < .05.


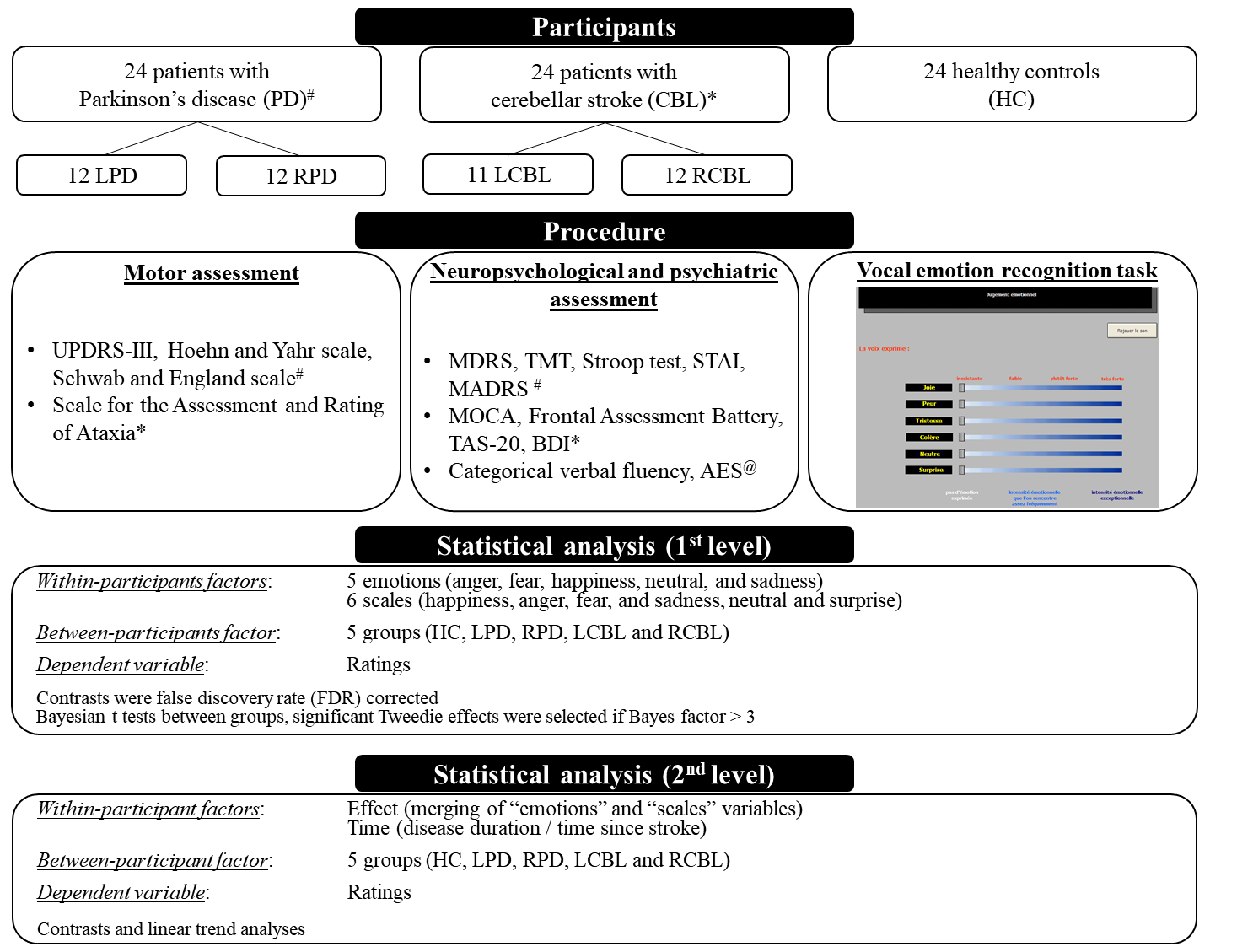


**Figure S1. Model design illustration of study method.**

*Note.* HC: healthy controls; LCBL: patients with left cerebellar stroke; LPD: patients with Parkinson’s disease exhibiting predominantly left-sided motor symptoms; RCBL: patients with right cerebellar stroke; RPD: patients with Parkinson's disease exhibiting predominantly right-sided motor symptoms. ^#^ Assessment performed only in patients with PD; * Assessment performed only in patients with CBL; ^@^ Assessment performed in both groups of patients.
